# Supplementary material for: Do Consumers Value Welfare and Environmental Attributes in Egg Production Similarly in Fresh Eggs and Prepared Meals?
Source: Animals (Basel). 2023 Jan 17;13(3):324. doi: 10.3390/ani13030324 (PMC9913460; doi:10.3390/ani13030324)
Supplement: Supplementary file 1 [file animals-13-00324-s001.zip › animals-2041737-supplementary-1.pdf]

SCREENING

Premièrement, nous avons quelques brèves questions pour vérifier que cette étude vous concerne.

[âge suivant question standard]

[sexe suivant question standard]

QD. Lequel des énoncés suivants vous décrit le mieux ? (Veuillez n'en choisir qu'un)

❑ Je suis la principale personne responsable des achats alimentaires au sein de mon ménage
❑ Je fais partie des personnes responsables des achats alimentaires au sein de mon ménage
❑ Je n’ai pas la responsabilité de faire les achats alimentaires au sein de mon ménage

[SI 3 - REMERCIEZ ET TERMINEZ.]

QD2. Combien de **douzaines d’œufs** votre ménage utilise-t-il ou consomme-t-il en moyenne par mois ?
❑ Plus de 8 douzaines d’œufs par mois
❑ 4 à 8 douzaines d’œufs par mois
❑ 1 à 3 douzaines d’œufs par mois
❑ Moins d’une douzaine d’oeufs par mois
❑ Nous ne consommons pas d’œufs du tout

[SI 5 - REMERCIEZ ET TERMINEZ.]

[DEBUT DE LA PREMIERE PARTIE COMMUNE A TOUS LES TRAITEMENTS]

INTRODUCTION

Dans le cadre d'une étude pilote financée par l'Institut sur la nutrition et les aliments fonctionnels (INAF), des chercheurs du département d'économie agricole et des sciences de la consommation de l'Université Laval entreprennent une étude sur la perception des consommateurs à l'égard de différentes catégories d'aliments. Ce sondage en ligne ne devrait pas prendre plus de 15 minutes.

Tout d’abord, voici quelques informations générales que nous vous demandons de lire, au sujet des mots que le questionnaire va employer :

<u>Poules en liberté</u> : les poules sont en très grand groupe et se déplacent librement dans le poulailler, elles ont accès à un perchoir et à un endroit (nichoir) pour pondre leurs œufs.

<u>Poules en cage</u> : les poules sont en très petit groupe et leurs déplacements sont limités à la surface de leur cage. Elles n’ont pas accès à un endroit spécifique pour pondre leurs œufs.

<u>Poules en logement aménagé</u> : les poules sont en petit groupe et peuvent se déplacer à l’intérieur d’un espace restreint, elles ont accès à un perchoir et à un endroit (nichoir) pour pondre leurs œufs.

<u>Poules en libre parcours</u> : les poules sont en très grand groupe et se déplacent librement dans le poulailler, elles ont accès à un perchoir et à un endroit (nichoir) pour pondre leurs œufs. Lorsque la température le permet, elles ont aussi accès à un enclos extérieur.

Nous allons à présent vous présenter des choix de produits alimentaires. Imaginez que vous êtes en train de faire vos courses à l'épicerie.

[RANDOMISER L'ORDRE D'APPARITION DES BLOCS DE PRODUITS : œufs, quiche, salade]

Vous vous trouvez au rayon des **œufs**. Merci d'indiquer votre choix face aux options suivantes pour une **boîte de 12 œufs de gros calibre de marque nationale** :

[RANDOMISER L'ORDRE DES COLONNES]

Œufs de poules en liberté	Œufs de poules en logement aménagé	Œufs de poules élevées en libre parcours	Œufs de poules en cage
5,89$	4,10 $	6,10$	3,59$
◯ je choisis ce produit	◯ je choisis ce produit	◯ je choisis ce produit	◯ je choisis ce produit

Vous vous trouvez au rayon des **quiches**. Merci d'indiquer votre choix face aux options suivantes pour une **quiche légumes et fromages de 575 grammes de marque nationale** :

[RANDOMISER L'ORDRE DES COLONNES]

Quiche préparée avec des œufs de poules en cage	Quiche préparée avec des œufs de poules en logement aménagé	Quiche préparée avec des œufs de poules en libre parcours	Quiche préparée avec des œufs de poules en liberté
7,99$	8,39$	9,99$	9,49$
◯ je choisis ce produit	◯ je choisis ce produit	◯ je choisis ce produit	◯ je choisis ce produit

Vous vous trouvez au rayon des **salades prêtes à manger**. Merci d'indiquer votre choix face aux options suivantes pour une **salade œufs-épinards (œufs, épinards, champignons) de 200 grammes** :

[RANDOMISER L'ORDRE DES COLONNES]

Salade composée avec des œufs de poules en cage	Salade composée avec des œufs de poules en logement aménagé	Salade composée avec des œufs de poules en élevage libre parcours	Salade composée avec des œufs de poules en liberté
5,49$	5,59$	5,85$	5,75$
◯ je choisis ce produit	◯ je choisis ce produit	◯ je choisis ce produit	◯ je choisis ce produit

[FIN DE LA PREMIERE PARTIE COMMUNE A TOUS LES TRAITEMENTS]

[Pour la suite du questionnaire, le participant est affecté aléatoirement, et uniquement à l'un des trois traitements : soit Bien-être animal, soit nutrition, soit environnement]

[DEBUT DE LA PARTIE SPECIFIQUE AU TRAITEMENT SUR LE BIEN ÊTRE ANIMAL]

Score de bien-être animal

Un score de bien-être animal peut être calculé pour des produits alimentaires à base de produits animaux. Pour chacun des produits présentés précédemment, nous avons donc calculé un score de bien-être animal.

Pour calculer ce score dans le cas des produits à base d'œufs, chaque type de logement est évalué selon une liste de critères comprenant des éléments relatifs aux moyens donnant aux poules la capacité d'exprimer leur comportement naturel, mais également des critères de santé (fractures, picage), des critères d'hygiène, de sécurité sanitaire, de qualité de l'air et d'impacts environnementaux de l'élevage.

Ce calcul s'appuie sur une méthodologie développée par des experts scientifiques indépendants.

Ce score donne donc une information globale évaluant les conditions de vie de la poule pondeuse ainsi que l'hygiène et la salubrité des œufs. Il tient compte d'arbitrages entre différentes dimensions du bien-être. Aucun système, en effet, n'est idéal selon tous les facteurs, et chacun comporte des compromis non seulement en ce qui concerne la santé de la poule, mais également en ce qui concerne la santé humaine, la pollution de l'environnement et les coûts de production des œufs.

Selon le score obtenu, chaque aliment ou breuvage se voit attribuer une lettre variant de A (niveau le plus élevé de bien-être) à E (niveau le plus bas).

Score de bien-être animal :

Bien-être plus élevé	A	B	C	D	E	*Bien-être moins élevé*

Nous allons à présent vous présenter des choix de produits alimentaires. Imaginez que vous êtes en train de faire vos courses à l'épicerie.

[RANDOMISER L'ORDRE D'APPARITION DES BLOCS DE PRODUITS : œufs, quiche, salade]

Vous vous trouvez au rayon des **œufs**. Merci d'indiquer votre choix face aux options suivantes pour une **boîte de 12 œufs de gros calibre de marque nationale** :

[RANDOMISER L'ORDRE DES COLONNES]

Produit	Œufs de poules en liberté	Œufs de poules en logement aménagé	Œufs de poules élevées en libre parcours	Œufs réguliers de poules en cage
Prix	5,89$	4,10 $	6,10$	3,59$
Score de bien-être animal	C	A	B	D
Votre choix ?	◯ je choisis ce produit	◯ je choisis ce produit	◯ je choisis ce produit	◯ je choisis ce produit

Vous vous trouvez au rayon des **quiches**. Merci d'indiquer votre choix face aux options suivantes pour une **quiche légumes et fromages de 575 grammes de marque nationale** :

[RANDOMISER L'ORDRE DES COLONNES]

Produit	Quiche préparée avec des œufs de poules en cage	Quiche préparée avec des œufs de poules en logement aménagé	Quiche préparée avec des œufs de poules en libre parcours	Quiche préparée avec des œufs de poules en liberté
Prix	7,99$	8,39$	9,99$	9,49$
Score de bien-être animal	D	A	B	C
Votre choix ?	◯ je choisis ce produit	◯ je choisis ce produit	◯ je choisis ce produit	◯ je choisis ce produit

Vous vous trouvez au rayon des **salades prêtes à manger**. Merci d'indiquer votre choix face aux options suivantes pour une **salade œufs-épinards (œufs, épinards, champignons) de 200 grammes** :

[RANDOMISER L'ORDRE DES COLONNES]

Produit	Salade composée avec des œufs de poules en cage	Salade composée avec des œufs de poules en logement aménagé	Salade composée avec des œufs de poules en libre parcours	Salade composée avec des œufs de poules en liberté
Prix	5,49$	5,59$	5,85$	5,75$
Score de bien-être animal	D	A	B	C

Votre choix ?	◯ je choisis ce produit	◯ je choisis ce produit	◯ je choisis ce produit	◯ je choisis ce produit

[FIN DE LA PARTIE SPECIFIQUE AU TRAITEMENT SUR LE BIEN ÊTRE ANIMAL]

[DEBUT DE LA PARTIE SPECIFIQUE AU TRAITEMENT SUR LA NUTRITION]

Score global de la qualité nutritionnelle des produits alimentaires

Un score de qualité nutritionnelle peut être calculé pour les produits alimentaires et les breuvages. Ce score a été calculé pour chacun des produits présentés précédemment.

Pour calculer ce score, on effectue un profilage nutritionnel, qui permet de caractériser la qualité nutritionnelle « globale » du produit en tenant compte de son contenu en plusieurs nutriments ou ingrédients d'intérêt publique (p. ex. sucres, sodium, fibres, grains entiers) plutôt qu'en se concentrant sur leur teneur en un seul nutriment ou ingrédient.

Ce calcul s'appuie sur une méthodologie développée par des experts scientifiques indépendants.

Cet outil génère un score global à partir de points attribués à un aliment ou breuvage en fonction de son contenu en quatre nutriments « à limiter » (énergie, gras saturés, sucres, sodium) ainsi qu'en trois nutriments ou composantes « à encourager » (fibres, protéines et proportion d'ingrédients d'origine végétale, comme les fruits et légumes).

Selon le score obtenu, chaque aliment ou breuvage se voit attribuer une lettre variant de A (meilleure qualité nutritionnelle) à E (plus faible qualité nutritionnelle).

Score de qualité nutritionnelle :

Qualité nutritionnelle plus élevée	A	B	C	D	E	*Qualité nutritionnelle plus faible*

Nous allons à présent vous présenter des choix de produits alimentaires. Imaginez que vous êtes en train de faire vos courses à l'épicerie.

[RANDOMISER L'ORDRE D'APPARITION DES BLOCS DE PRODUITS : œufs, quiche, salade]

Vous vous trouvez au rayon des **œufs**. Merci d'indiquer votre choix face aux options suivantes pour une **boîte de 12 œufs de gros calibre de marque nationale** :

[RANDOMISER L'ORDRE DES COLONNES]

Produit	Œufs de poules en liberté	Œufs de poules en logement aménagé	Œufs de poules élevées en libre parcours	Œufs réguliers de poules en cage
Prix	5,89$	4,10 $	6,10$	3,59$
Score de qualité nutritionnelle	A	A	A	A
Votre choix ?	◯ je choisis ce produit	◯ je choisis ce produit	◯ je choisis ce produit	◯ je choisis ce produit

Vous vous trouvez au rayon des **quiches**. Merci d'indiquer votre choix face aux options suivantes pour une **quiche légumes et fromages de 575 grammes de marque nationale** :

[RANDOMISER L'ORDRE DES COLONNES]

Produit	Quiche préparée avec des œufs de poules en cage	Quiche préparée avec des œufs de poules en logement aménagé	Quiche préparée avec des œufs de poules en libre parcours	Quiche préparée avec des œufs de poules en liberté
Prix	7,99$	8,39$	9,99$	9,49$
Score de qualité nutritionnelle	C	C	C	C
Votre choix ?	◯ je choisis ce produit	◯ je choisis ce produit	◯ je choisis ce produit	◯ je choisis ce produit

Vous vous trouvez au rayon des **salades prêtes à manger**. Merci d'indiquer votre choix face aux options suivantes pour une **salade œufs-épinards (œufs, épinards, champignons) de 200 grammes** :

[RANDOMISER L'ORDRE DES COLONNES]

Produit	Salade composée avec des œufs de poules en cage	Salade composée avec des œufs de poules en logement aménagé	Salade composée avec des œufs de poules en libre parcours	Salade composée avec des œufs de poules en liberté
Prix	5,49$	5,59$	5,85$	5,75$
Score de qualité nutritionnelle	B	B	B	B
Votre choix ?	◯ je choisis ce produit	◯ je choisis ce produit	◯ je choisis ce produit	◯ je choisis ce produit

[FIN DE LA PARTIE SPECIFIQUE AU TRAITEMENT SUR LA NUTRITION]

[DEBUT DE LA PARTIE SPECIFIQUE AU TRAITEMENT SUR L'ENVIRONNEMENT]

Score de qualité environnementale

Un score de qualité environnementale peut être calculé pour les produits alimentaires et les breuvages. Ce score a été calculé pour chacun des produits présentés précédemment.

Pour calculer ce score, on évalue le mode de production de chaque ingrédient, selon une analyse de cycle de vie (ACV) qui se base sur la proportion des ingrédients dans chaque produit.

Ce calcul s'appuie sur une méthodologie développée par des experts scientifiques indépendants.

Le score fournit une vision globale considérant les émissions de gaz à effets de serre de multiples impacts environnementaux.

Selon le score obtenu, chaque aliment ou breuvage se voit attribuer une lettre variant de A (meilleure qualité environnementale) à E (plus faible qualité environnementale).

Score de qualité environnementale :

Qualité environnementale plus élevée	A	B	C	D	E	*Qualité environnementale plus faible*

Nous allons à présent vous présenter des choix de produits alimentaires. Imaginez que vous êtes en train de faire vos courses à l'épicerie.

Vous vous trouvez au rayon des **œufs**. Merci d'indiquer votre choix face aux options suivantes pour une **boîte de 12 œufs de gros calibre de marque nationale** :

[

Produit	Œufs de poules en liberté	Œufs de poules en logement aménagé	Œufs de poules élevées en libre parcours	Œufs réguliers de poules en cage
Prix	5,89$	4,10 $	6,10$	3,59$
Score de qualité environnementale	C	B	C	B
Votre choix ?	◯ je choisis ce produit	◯ je choisis ce produit	◯ je choisis ce produit	◯ je choisis ce produit

Vous vous trouvez au rayon des **quiches**. Merci d'indiquer votre choix face aux options suivantes pour une **quiche légumes et fromages de 575 grammes de marque nationale** :

Produit	Quiche préparée avec des œufs de poules en cage	Quiche préparée avec des œufs de poules en logement aménagé	Quiche préparée avec des œufs de poules en libre parcours	Quiche préparée avec des œufs de poules en liberté
Prix	7,99$	8,39$	9,99$	9,49$
Score de qualité environnementale	A	A	B	B
Votre choix ?	◯ je choisis ce produit	◯ je choisis ce produit	◯ je choisis ce produit	◯ je choisis ce produit

Vous vous trouvez au rayon des **salades prêtes à manger**. Merci d'indiquer votre choix face aux options suivantes pour une **salade œufs-épinards (œufs, épinards, champignons) de 200 grammes** :

Produit	Salade composée avec des œufs de poules en cage	Salade composée avec des œufs de poules en logement aménagé	Salade composée avec des œufs de poules en libre parcours	Salade composée avec des œufs de poules en liberté
Prix	5,49$	5,59$	5,85$	5,75$
Score de qualité environnementale	A	A	B	B
Votre choix ?	◯ je choisis ce produit	◯ je choisis ce produit	◯ je choisis ce produit	◯ je choisis ce produit

[FIN DE LA PARTIE SPECIFIQUE AU TRAITEMENT SUR L'ENVIRONNEMENT]

[DEBUT DE LA SECONDE PARTIE COMMUNE A TOUS LES TRAITEMENTS]

Pour terminer, quelques questions sur vous…

[niveau d'éducation suivant question standard LM]

Dans quelle mesure êtes-vous d'accord ou en désaccord avec les énoncés figurant dans le tableau ci-dessous? (1 = fortement en désaccord, 2 = en désaccord, 3 = indifférent, 4 = d'accord, 5 = fortement d'accord) [RANDOMISER LES ITEMS DU TABLEAU]

	1	2	3	4	5
Quand je mange sainement, j'ai le sentiment de prendre soin de moi					
J'ai du plaisir à bien manger, et le fait que ça soit bon pour la santé y contribue					
Après avoir mangé sainement je ressens un bien-être					
Le plaisir de manger sainement va de pair avec le plaisir de prendre le temps de choisir les bons ingrédients					
J'aime me faire du bien en mangeant sainement					
Je m'inquiète de ma santé					
Veillez sélectionner « en désaccord » pour cette ligne					

Dans quelle mesure êtes-vous d'accord ou en désaccord avec les énoncés figurant dans le tableau ci-dessous? (1 = fortement en désaccord, 2 = en désaccord, 3 = indifférent, 4 = d'accord, 5 = fortement d'accord) [RANDOMISER LES ITEMS DU TABLEAU]

	1	2	3	4	5
Il est important pour moi d'avoir une alimentation équilibrée					
Il est important pour moi de manger sainement pour être plus en forme					
Il est important pour moi que mon alimentation me procure une quantité suffisante de vitamines et de minéraux.					
Je vérifie habituellement les ingrédients des produits alimentaires sur les étiquettes.					
Je passe beaucoup de temps au supermarché à lire l'information sur la nutrition					
Je suis préoccupé par l'utilisation des antibiotiques et les pesticides dans le processus de production					

Il est important pour moi que les aliments que je mange ne contiennent pas d'additifs ou d'agents de conservations					
Je pense qu'il y a un lien direct entre l'alimentation et la santé					
Manger sainement contribue à réduire les risques de maladies reliées à l'obésité et cardiovasculaires					
Je pense qu'il y a un lien direct entre l'alimentation et la prévention de différentes formes de cancer.					
Je pense que l'alimentation des animaux a un fort impact sur le goût de la viande, du lait et des œufs					
Je pense que l'alimentation des animaux a un fort impact sur la qualité nutritionnelle de la viande, du lait et des œufs					
Je pense que le mode de culture (biologique, conventionnel, raisonné…) a un fort impact sur la qualité nutritionnelle des légumes et des fruits					
Je pense que le mode de culture (biologique, conventionnel, raisonné…) a un fort impact sur la qualité nutritionnelle des céréales					
Je pense que le mode de culture (biologique, conventionnel, raisonné…) a un fort impact sur le goût des légumes et des fruits					
Je pense que le mode de culture (biologique, conventionnel, raisonné…) a un fort impact sur le goût des céréales					
Je pense que l'utilisation d'additifs ou de conservateurs a un fort impact sur le goût des aliments					
Je pense que l'utilisation d'additifs ou de conservateurs a un fort impact sur la qualité nutritionnelle des aliments					

Pourriez-vous nous indiquer votre niveau de préoccupation concernant : [RANDOMISER LES ITEMS DU TABLEAU]

	Très faible	Faible	Moyen	Fort	Très fort
Votre santé?					
L'environnement?					
Le bien être animal?					

[FIN DE LA SECONDE PARTIE COMMUNE A TOUS LES TRAITEMENTS]
